# Design and test of an automated version of the modified Jebsen test of hand function using Microsoft Kinect

**DOI:** 10.1186/s12984-017-0250-1

**Published:** 2017-05-02

**Authors:** Daniel Simonsen, Ida F. Nielsen, Erika G. Spaich, Ole K. Andersen

**Affiliations:** Integrative Neuroscience group, SMI®, Department of Health Science and Technology, Aalborg University, Aalborg, Denmark

**Keywords:** Stroke, Microsoft Kinect, Hand function, Motor function test

## Abstract

**Background:**

The present paper describes the design and evaluation of an automated version of the Modified Jebsen Test of Hand Function (MJT) based on the Microsoft Kinect sensor.

**Methods:**

The MJT was administered twice to 11 chronic stroke subjects with varying degrees of hand function deficits. The test times of the MJT were evaluated manually by a therapist using a stopwatch, and automatically using the Microsoft Kinect sensor. The ground truth times were assessed based on inspection of the video-recordings. The agreement between the methods was evaluated along with the test-retest performance.

**Results:**

The results from Bland-Altman analysis showed better agreement between the ground truth times and the automatic MJT time evaluations compared to the agreement between the ground truth times and the times estimated by the therapist. The results from the test-retest performance showed that the subjects significantly improved their performance in several subtests of the MJT, indicating a practice effect.

**Conclusions:**

The results from the test showed that the Kinect can be used for automating the MJT.

## Background

Deficits in motor function, in the form of hemiparesis or hemiplegia, are a frequent consequence of cerebral stroke [1]. Even though motor function may be regained to some extent through intensive rehabilitative training following acute treatment of stroke, deficits in hand function often remain [2, 3]. Following discharge from the rehabilitation unit, patients are typically asked to perform unsupervised self-training in their own home. The lack of supervision during training at home will likely have an impact on the patient’s training compliance and training quality. Therefore, it is important to perform regular evaluations of the patient’s functional level in order to provide useful supervision and to maintain patient motivation. The patients’ performance in a specific motor function test provides valuable insight into whether the training scheme chosen for a patient is effective or it should be changed. Thus, it is very important that the motor function tests being used are objective and reflect the actual functional level of the patient being tested. Several validated motor function tests including assessment of hand function exist, e.g. Jebsen Test of Hand Function [4], Action Research Arm Test [5], Fugl-Meyer Assessment [6], Wolf Motor Function Test (WMFT) [7], Box and Blocks Test [8] and Nine Hole Peg Test [9]. Common for all these tests is that they must be administered by a therapist, which might be a source for variability in the test results, and cause the test results not always to be completely reproducible and objective. In tests including performance time as an outcome measure, e.g. the WMFT, the reaction time of the subject could introduce a bias to the results, as suggested by previous studies [10, 11]. Likewise, the end time of the test would likely be subjected to a bias, since the examiner has a finite reaction time. Thus, both the reaction time of the examiner and the subject could be potential sources of bias and variability in timed motor function tests. The sensitivity of a motor function test is affected by sources of bias and variability and therefore it is of interest to minimize these, to make detection of even small changes possible.

By automating motor function tests, the objectivity of the tests would be increased. This might also make possible to use the tests at remote sites, without direct supervision, as a part of a tele-rehabilitation service. Finally, automated tests could be administered more frequently. Previous studies have shown that selected parts of the WMFT can be automated by use of motion sensors mounted on the body of healthy subjects [10] and stroke patients [11]. Both systems automated the test by analyzing three-dimensional kinematics data from body-worn sensors (inertial measurement units) mounted on the most affected wrist, arm and shoulder of stroke patients [10, 11]. Similarly, using inertial measurement unit sensors, Yang et al. (2013) showed that when administering the 10 m walking test, the output from their system was in close agreement with the walking speeds estimated using a stop-watch [12]. These systems require though correct positioning and mounting of the motion sensors [10]. Huang et al. (2012) showed that also a computer vision based approach, consisting of a monitor camera and a Xilinx Virtex II Pro Field Programmable Gate Array (for computation), may be used for automating the WMFT. All participants being tested had to wear a black sweatband on the wrist of the extremity being tested [13]. Another low-price method for capturing the movements of a patient performing a motor function test is the Microsoft Kinect sensor (Kinect). By using a Kinect, the need for body mounted sensors is eliminated, thus lowering the susceptibility to data loss and easing donning and doffing of the system. Furthermore, the Microsoft Kinect sensor is a low-cost commercially available device. In this paper, we describe the design and test of a Kinect based system for automatic evaluation of a standardized, validated motor function test, administered to stroke patients with hand function deficits. The Modified Jebsen Test of Hand Function (MJT) [14], initially proposed by Bovend’Eerdt et al. (2004) as a test for assessment of gross functional dexterity in stroke patients, was selected for automation as this test is easy to administer and takes short time to complete.

## Methods

### Subjects

The MJT was administered twice by the same therapist to 11 chronic stroke subjects (verified by magnetic resonance imaging scans) with varying degrees of hand function deficits, and aged between 50 and 80 years (Table 1).

**Table 1:** Subject age, gender (F: female, M: male), most affected hand (D: dominant hand, N: non-dominant hand), test scores from Bergs Balance Scale (BBS), and Six Minutes Walk Test (SMWT)

	Age	Gender	Most affected hand	Stroke type	Time since stroke	BBS	SMWT
P1	80	F	N	Hemorrhagic	5 m	-	410 m
P2	77	F	D	Ischemic	5 m	-	397 m
P3	72	M	D	Hemorrhagic	5 m	36/56	169 m
P4	53	M	D	Ischemic	5 m	36/56	158 m
P5	79	M	N	Ischemic	5 m	-	-
P6	70	F	N	Ischemic	1 m	-	-
P7	58	F	D	Ischemic	2 m	-	-
P8	76	M	D	Ischemic	2 m	-	-
P9	66	M	N	Hemorrhagic	5 m	-	100 m
P10	77	M	D	Ischemic	1 m	-	-
P11	53	M	D	Ischemic	2 m	54/56	610 m

All tests were carried out at a local rehabilitation unit. Signed consent was obtained from all subjects prior to the experiment and the Declaration of Helsinki was respected. The study was approved by the local ethical committee (approval no. N-20130053).

### Modified Jebsen test of hand function

**Fig. 1:**
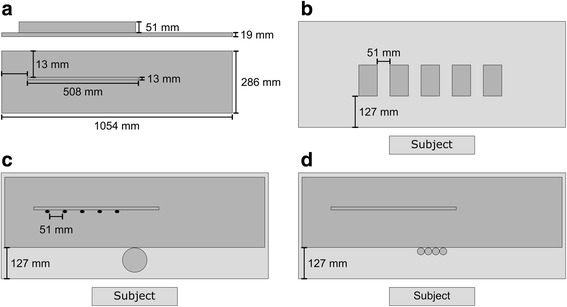
**a**) shows the wooden board used in the MJT seen from the side and from above. The three other quarters of the figure show a schematic layout of each of the three subtests of the MJT **b**) Card Turning, **c**) Simulated Feeding, **d**) Stacking Checkers)

The MJT consisted of three timed subtests, which were carried out by the subject, seated in front of a table. First, the most affected hand was tested and then, the least affected hand was tested. The items used in the test were part of the Jebsen Test of Hand Function test kit from Patterson Medical Ltd. [15], all identical in shape and size to the items described by Jebsen et al. [4]. The following items from the test kit were used in the MJT:

- Five white cardboard cards with green markers (L: 76 mm, W: 127 mm, H: 1 mm)
- Five dried kidney beans (approximately L: 15 mm, W: 6 mm, H: 6 mm)
- An open can (H: 100 mm, Ø: 100 mm)
- A teaspoon
- A wooden board (L: 1054 mm, W: 286 mm, H: 19 mm) with a ridge (L: 508 mm, W: 13 mm, H: 51 mm. Fig. 1).
- Four red wooden checkers (H: 6 mm, Ø: 30 mm)
- A stop-watch

Prior to each subtest, the therapist instructed the subject in the procedure of the test and asked the subject to place his/her hand at the edge of the table. The therapist verbally signaled to the subject when the subtest was to start and concurrently started the stopwatch. The procedure for each subtest is described in the following sections.

1. Procedure for Card Turning
  Five cardboard cards were placed 51 mm apart in a horizontal row on the table (a green marker was stuck to each card, the side of the cards with the marker faced down), 127 mm from the front edge of the table (Fig. 1). The subject was instructed to turn around the cardboard cards one by one as quickly as possible. Timing ended when all cards had been turned around.
2. Procedure for Simulated Feeding
  The board was placed 127 mm from the front edge of the table. Five kidney beans were placed on the board touching the center ridge 51 mm apart. The open can was placed centrally in front of the board (grey filled circle, part c) of Fig. 1). The teaspoon was provided to the subject and the subject was instructed to pick up the beans one by one using the teaspoon and drop them into the can as quickly as possible. Timing ended when the fifth bean hit the bottom of the can.
3. Procedure for Stacking Checkers
  The board was placed 127 mm from the front edge of the table. The four checkers were placed in a horizontal row, side by side, centrally in front of and touching the board (Fig. 1). The subject was instructed to stack the checkers, one at a time, on top of each other, on top of the board. Timing ended when the third checker made contact with the fourth.

### System setup

A Microsoft Kinect sensor was used for capturing the subject performing the MJT. The Kinect sensor was mounted on a tripod placed next to the table, which was placed in a position where no ceiling lights were located exactly above it. The Kinect was positioned approximately 1200 mm above the center of the table for capturing the subjects’ movements during the subtests. The Kinect has a frame rate of 30 frames per second and a resolution of 640 × 480 pixels. Each captured frame consists of an 8 bit RGB (red, green, blue) and a 16 bit depth image, where each pixel contains the distance to the Kinect. Each Kinect recording was initiated before the therapist started the subtest and ended after the subtest was completed.

### Card turning – automatised detection methods

1. Overview
  The flowchart depicted on Fig. 2 provides an overview of the detection methods used for automating the Card Turning subtest.

**Fig. 2:**
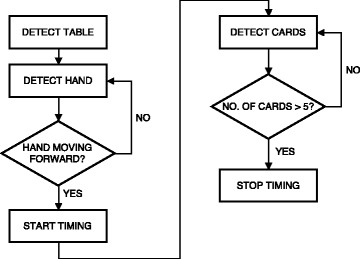
Flowchart describing the order of the detection methods and cues used by the detection methods during the Card Turning subtest
2. Table Detection
  Detection of the surface and the edge of the table was based on the analysis of the depth image. The depth level of the surface of the table was calculated as the median depth of all pixels, since the table was the main field of view. Then, a binary image was created by thresholding the depth image (upper) into two parts, one part containing all pixels with a depth value lower than a depth level of 300 mm below the surface of the table and the other part containing pixels with depth values above this threshold (Fig. 3). Finally, the edge of the table was found by detecting the location of the change of the binary image (median) in the proximal-distal direction, in the bottom half of the image.

**Fig. 3:**
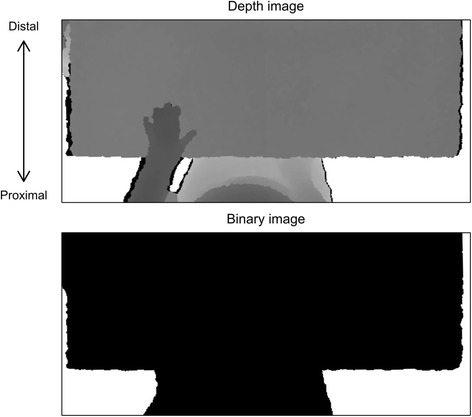
The edge of the table was detected in the binary image (*lower*) produced by thresholding the depth image (*upper*) into two parts, one part containing all pixels with a depth value *lower* than a depth level of 300 mm below the surface of the table and the other part containing pixels with depth values above this threshold
3. Hand Detection
  In each frame, the hand was detected as any group of 50 or more connected pixels (one pixel was considered to be connected to another pixel if it was located exactly on top of, below, left or right to the other pixel), corresponding to an area of approximately 10 mm^2^, located more than 30 mm above the table, and located inside the estimated area of the table in the depth image. The position of the distal part of the hand was computed as the centroid of the hand pixels located in the area between the hand pixel most distal to the edge of the table and no more than 10 mm from this pixel.
4. Card Detection
  Cards being turned were identified by detecting the green markers in the RGB image. All pixels having an R and B value lower than the corresponding G value were set to 1 and the remaining pixels to 0. A 2-dimensional 5-by-5 pixels median filter was then applied to the binary image. Groups of connected pixels consisting of less than 10 pixels (corresponding to an area of 3 mm^2^) were excluded, along with groups of pixels with a distance from its centroid to any point of the hand of less than 10 mm. The centroid of each of the remaining groups of connected pixels was saved for each frame. Centroids detected in 15 or more frames, located less than 5 mm from each other were classified as one individual card that had been turned.
5. Start and Finish Time Detection
  Detection of the start time was based on the analysis of the distance between the hand centroid and the edge of the table, which was located at 0 mm (Fig. 4). First, the moment when the distance exceeded 127 mm (distance at which the distal part of the hand crossed the proximal edge of the cards) was found; then, the movement onset, i.e. the start time, was determined as the time at which a minimum displacement of 3 mm/frame first happened before the hand reached the card.
  Detection of the finish time was based on the detections of the cards. Once five cards had been detected, all cards were considered to be turned and timing was stopped.

**Fig. 4:**
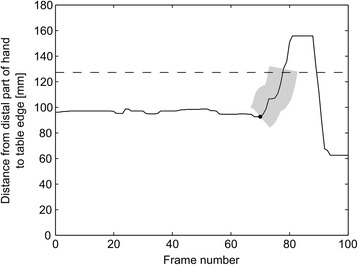
The figure shows the distance between the hand centroid and the edge of the table (located at 0 mm) for the frames recorded during one card turn in the Card Turning subtest. The *grey area* shows the displacement of the hand before exceeding 127 mm (*indicated by a dashed line*) having an initial increase of more than 3 mm/frame. The estimated starting time is marked with a *black dot*

### Simulated feeding – detection methods

1. Overview
  The flowchart depicted on Fig. 5 provides an overview of the detection methods used for automating the Simulated Feeding subtest.

**Fig. 5:**
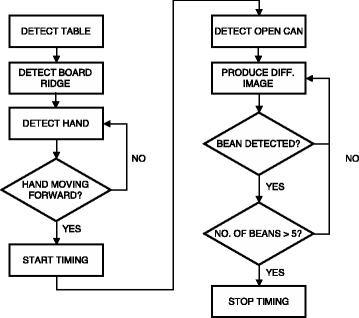
Flowchart describing the order of the detection methods and cues used by the detection methods during the Simulated Feeding subtest
2. Board Detection
  The board used in the Simulated Feeding and Stacking Checkers subtest (section F) was detected using the depth image. Initially, the board ridge was detected by dividing the depth image at a depth level 50 mm above the estimated surface of the table (the ridge was expected to be located at 70 mm (board thickness: 19 mm and ridge height: 51 mm)), thereby, separating the upper part of the ridge from the board and the surface of the table. The groups of connected pixels in the binary image which length fell within ± 20% of the length of the board ridge (508 mm) were considered part of the ridge. The position of the board was then estimated from the detected position of the ridge.
3. Hand Detection
  The detection of the hand in the current subtest was based on the method described in section D.2, but also depended on the position of the individual pixels of the hand. As in the previous method the hand was detected as any group of 50 or more connected pixels located more than 30 mm above the table, board or board ridge depending on the position of the individual pixels (Fig. 6). Additionally, the pixels had to be located inside the estimated area of the table in the depth image. The position of the distal part of the hand was computed using the method described in section D.2.

**Fig. 6:**
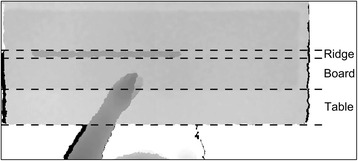
The method used for detecting the hand in the Simulated Feeding subtest depended on the location of the pixel analyzed with regards to the surface below (*ridge*, *board*, or *table*)
4. Open Can Detection
  For detection of the open can the RGB image was used. The location of the open can was estimated by applying the Circle Hough Transform [16] to the image subset in the first frame of the recording. In cases where multiple circles were detected, the diameter of each circle was compared to the diameter of the open can (100 mm). The position of the circle with the diameter closest to 100 mm was detected as the position of the open can.
5. Bean Detection
  The beans used in subtest 2 had to be detected inside the area enclosed by the open can. For each frame the image subset of the RGB image described in the previous section was converted to a gray scale image. A difference image was produced by calculating the absolute difference between the previous and current gray scale images (Fig. 7). Only pixels with a value above 10% of the full range (0–255) and pixels detected as belonging to the hand were included in the subsequent analysis.
  Groups of connected pixels with centroids located closer than 5 mm were considered as one single group, in order to smooth the difference image. A bean was detected as being dropped into the cylinder if a group of 5 to 50 connected pixels (corresponding to an area of 3.5-10 mm^2^) with a length less than 15 mm was found within the open can, and the distance from the hand to the edge of the open can was less than 50 mm. Following detection of a bean dropped into the cylinder, a new bean would not be detected before the distance between the hand and the board ridge was less than 30 mm.

**Fig. 7:**
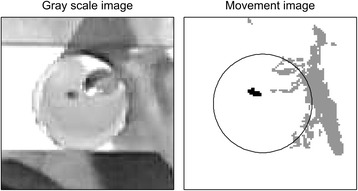
Difference image (*right side*) produced by calculating the absolute difference between two consecutive *gray scale* images (*left side*) were used for monitoring the activity within the open can. The *black colored* group of pixels on the right side image shows an example of a group of pixels detected as a bean dropping into the cylinder
6. Start and Finish Time Detection
  Detection of the start time was similar to the method used for start time detection in the card turning subtest. The method was based on analysis of the distance between the hand centroid and the edge of the table, which was located at 0 mm (Fig. 4). First, the moment when the distance exceeded 147 mm (distance where the distal part of the hand had moved 20 mm past the proximal edge of the board located 127 mm from the edge of the table) was found; then, the movement onset, i.e. the start time was determined as the time at which a minimum displacement of 3 mm/frame first happened before the hand had moved more than 20 mm past the proximal edge of the board.
  Timing was stopped when a total of five beans had been detected and the distance between the hand centroid and the edge of the open can exceeded 20 mm.

### Stacking checkers – detection methods

1. Overview
  The flowchart depicted on Fig. 8 provides an overview of the detection methods used for automating the Stacking Checkers subtest.

**Fig. 8:**
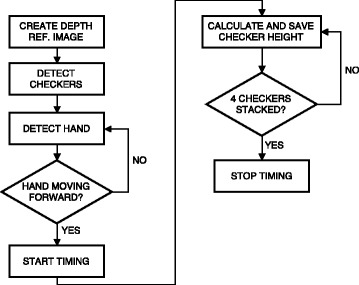
Flowchart describing the order of the detection methods and cues used by the detection methods during the Stacking Checkers subtest
2. Checker Detection
  To identify when the checkers were stacked, a depth reference image was produced by calculating the average depth value for each pixel for the initial 15 frames of the recording. In each of the following frames, the depth reference image was subtracted. All pixels with a distance larger than 5 mm to the detected surface of the board (Simulated feeding, board detection) were identified. Any group of connected pixels with a maximal length between 0.5 and 2 times the diameter of a checker, with its centroid located within the region of the board, and more than 30 mm from the pixels detected as the hand (Simulated feeding, hand detection) were detected as a checker. The average depth level of each detected checker was saved in a log along with the frame number.
3. Start and Finish Time Detection
  Detection of the start time was similar to the methods used for start time detection in the previous two subtests. It was based on analysis of the distance between the hand centroid and a point located 15 mm in front of the horizontal middle of the board edge, approximately between the two middle checkers (Fig. 4). First, the moment when the distance went below 63.5 mm (distance at which the distal part of the hand was no more than 3.5 mm from the right or left edge of the checkers) was found; then, the movement onset, i.e. the start time was determined as the time at which a minimum displacement of 3 mm/frame first happened before the hand reached the checkers.
  In each frame, the log of detected checkers was analyzed to determine whether all four checkers had been stacked. The number of checkers stacked was determined by sorting the depth level of all checker detections into four depth intervals: *(D ± 0.5∙D)∙n*, where *D* equals the height of one checker and *n* represents the number of checkers stacked, ranging from 1 to 4. The timing of the subtest was stopped, when five consecutive detections in the fourth interval had occurred (Fig. 9).

**Fig. 9:**
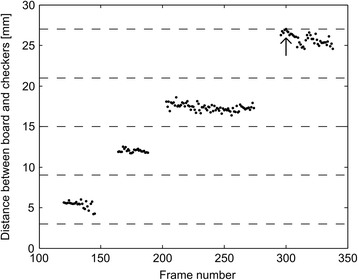
Example of the log data containing the depth level and frame number for each checker detected. The *dashed lines* represent the *upper* and *lower* boundaries of the four intervals used for determining the number of stacked checkers. The *arrow* indicates the finish time detection

### Ground truth test times – visual evaluation

The ground truth durations of all subtests were obtained by visually analyzing the RGB video recordings from the Kinect.

For the Card Turning subtest, the start time was found by identifying the frame where the subject started moving the hand from the starting position (at the edge of the table) towards the first card. The end time was found by identifying the frame where the subject placed the fifth, turned card on the table.

For the Simulated Feeding subtest, the start time was found by identifying the frame where the subject started moving the hand towards the position of the beans. The end time was found by identifying the frame where the fifth bean made contact with the bottom of the open can.

For the Stacking Checkers subtest, the start time was found by identifying the frame where the subject started moving the hand towards the position of the checkers. The end time was found by identifying the frame where all checkers were stacked and there was no visual contact between the checkers and the hand.

### Statistical analysis

1. Comparison of Measurement Methods for the Modified Jebsen Test of Hand Function
  The results from each subtest, for the most and least affected hand, measured by the therapist and the Kinect were compared using Bland-Altman analysis. Furthermore, each of these methods were compared to the ground truth results using also Bland-Altman analysis. This analysis includes calculation of the mean difference, referred to as the bias, between the two methods being compared along with the 95% limits of agreement (LoA) calculated as the mean difference ± 1.96 times the standard deviation of the differences. The true value for each subject was expected to vary from the first to the second repetition of the MJT due to a practice effect. Therefore, the calculations of the bias and limits of agreement were based on the method described in section 3 of the paper by Bland and Altman [17]. As the data was found to be normally distributed, t-tests, corrected for multiple comparisons (Bonferroni), were used to assess if the bias of each pair of compared methods was significantly different from 0 (significance level at *p* < 0.05). Equality of variances for the three methods comparisons were tested using Levenes test (results from the two sessions were pooled), corrected for multiple comparisons (significance level at *p* < 0.05). All values are reported as the value ± one standard deviation.
2. Test-retest Performance
  The results from the first and second repetition of each subtest for the most affected and the least affected hand measured by the therapist, Kinect, and the ground truth were compared using Wilcoxon signed rank tests (as the data was found not to be normally distributed), corrected for multiple comparisons (Bonferroni), (significance level at *p* < 0.05). The results from these tests are reported as the median (interquartile range).

## Results

### Comparison of measurement methods for the modified Jebsen test of hand function

**Fig. 10:**
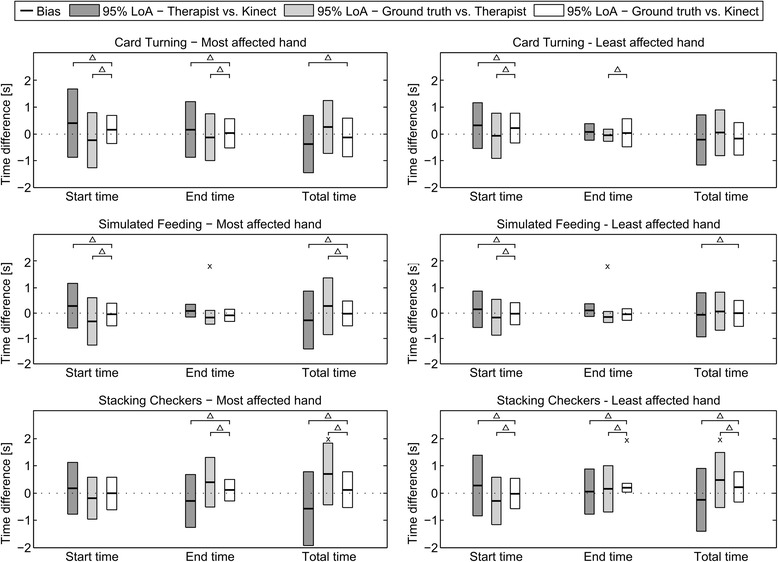
The figure provides an overview of the Bland-Altman analysis of the results from each subtest for the most and least affected hand. The results from each subtest measured by the therapist and the Kinect were compared and each of these methods was compared to the ground truth. Each *bar* shows the 95% LoA of the comparison and the thick, *black horizontal line* in the middle of the *bars* shows the bias. “Δ” indicates a statistical significant difference between the variances of two methods (Levene’s test) and “x” indicates a statistical significant bias

Figure 10 shows a graphical overview of the Bland-Altman analysis of the start, end, and total time of the results obtained by the three methods (therapist, Kinect and ground truth).

The width of the 95% LoA for the differences between the measurements performed by the therapist and Kinect of the start time, end time, and total time of each subtest ranged from 0.51 to 2.71 s.

Across all three subtests, the width of the 95% LoA of the differences between the measurements by the ground truth and Kinect were lower than the 95% LoA of the differences between the measurements between the ground truth and the therapist for the start time and total time. Furthermore, the analysis of the variance of the differences between the three methods, visualized on Fig. 10, showed that the variance of the difference between the ground truth and Kinect was always equal to or less than the variance of the differences between ground truth and the therapist, except in one case. The variance of the differences between the ground truth and end times detected by Kinect was less than the variance of the differences between the ground truth and the therapist. These results indicate that the precision of the estimates of the start and total time produced by Kinect was higher than the precision of the same estimates produced by the therapist.

None of the biases of the differences between the start times detected by the three different methods were statistically significant. Three biases of the differences between the end times detected by the different methods were statistically significant: the bias of the end times for ground truth vs. therapist for the Simulated Feeding subtest using the most affected hand (−0.18 ± 0.04 s, *p* = 0.002, *t* = 2.23) and the least affected hand (−0.17 s ± 0.04 s, *p* = 0.001, *t* = 2.23), and the bias of end times for ground truth vs. Kinect for the Stacking Checkers subtest using the least affected hand (0.20 s ± 0.04 s, *p* = 0.001, *t* = 2.23). Two of the biases of the differences between the ground truth and total times detected by the three methods were statistically significant: the bias of the ground truth vs. therapist for the Stacking Checkers subtest using the most affected hand (0.70 s ± 0.19 s, *p* = 0.004, *t* = 2.26) and the least affected hand (0.48 s ± 0.15 s, *p* = 0.01, *t* = 2.23).

### Test-retest performance

Tables 2 and 3 show the median time difference for each subtest for the most and least affected hand. The results from the MJT administered to the most affected hand measured by each method showed that the completion time of the second repetition of all subtests was significantly shorter compared to the first repetition (Table 2). In the Card Turning subtest all three methods detected statistically significant differences in completion times, whereas only one method detected a significant difference in completion time for the Simulated Feeding subtest (Therapist) and the Stacking Checkers subtest (Kinect).

**Table 2:** Median ground truth test times from the first test (MGT) and median time difference between the first and second repetition of the MJT administered to the most affected hand. Statistically significant results (*p* < 0.05) are followed by an asterisk

	MGT	Δ Therapist	Δ Kinect	Δ Ground truth
Card Turning	7.9 s	2.50 s (2.85 s)*	2.21 s (2.86 s)*	2.42 s (2.77 s)*
Simulated Feeding	10.7 s	1.90 s (4.27 s)*	2.47 s (4.67 s)	2.73 s (4.71 s)
Stacking Checkers	7.5 s	1.15 s (6.50 s)	1.12 s (6.22 s)*	1.48 s (6.12 s)

**Table 3:** Median ground truth test times from the first test (MGT) and median time difference between the first and second repetition of the MJT administered to the least affected hand. Statistically significant results (*p* < 0.05) are followed by an asterisk

	MGT	Δ Therapist	Δ Kinect	Δ Ground truth
Card Turning	6.7 s	1.00 s (1.35 s)*	1.09 s (1.03 s)*	1.17 s (1.08 s)*
Simulated Feeding	9.7 s	1.10 s (2.33 s)	0.81 s (2.42 s)	0.87 s (2.21 s)
Stacking Checkers	6.3 s	0.60 s (0.65 s)	0.66 s (1.04 s)	0.65 s (1.19 s)

The results from the MJT administered to the least affected hand measured by all methods showed that the completion time of the second repetition of the Card Turning subtest was significantly shorter compared to the first repetition (Table 3). No significant differences in completion times were detected by any of the three methods for the two other subtests.

## Discussion

In the present study an automated version of the Modified Jebsen Test of Hand Function based on a Kinect sensor was presented and tested. An occupational therapist administered the MJT twice to 11 stroke subjects with varying degrees of hand function deficits. The Kinect sensor was used for capturing the movements of the subjects during the test. The results showed that it was possible to automate the MJT with a Kinect sensor and the MJT evaluations produced using the Kinect were comparable to those obtained by the therapist. Generally, in the agreement analysis, the ground truth times and the Kinect times were in better agreement than the ground truth times and therapist times. The results obtained by the therapist and the Kinect showed that the patients significantly improved their performance in several different subtests of the MJT from the first to the second repetition, which indicates a practice effect.

### Comparison of methods for evaluation of the modified Jebsen test of hand function

The current study compared MJT results assessed manually by a therapist and automatically by use of a Kinect sensor. The two methods were also compared against the ground truth times. The Bland-Altman analysis showed that the width of the LoA’s of the therapist and Kinect times was up to 2.71 s, indicating poor agreement between the two methods. Comparing this result to the width of the LoA’s of the ground truth and therapist times (up to 2.26 s) and the width of the LoA’s of the ground truth and the Kinect times (1.43 s), it was clear that the poor agreement between the therapist and Kinect times was mainly due to a lack of precision in the therapist times. This imprecision of the therapist times might be explained partially by the reaction time of both the patient (start timing) and the therapist (end timing), which can be expected to be at least 0.35 s [18]. The main factor affecting the precision of the Kinect times is related to the detection methods, e.g. if certain parts of the image could not be detected near the end of a subtest due to occlusion of the visual contact between the Kinect and the object, the estimate of the end time is affected. A different positioning of the Kinect sensor, e.g. non-perpendicular to the table surface or use of more than one Kinect sensor, might have been able to prevent visual occlusion. Start times estimated by the Kinect were in risk of being detected too early, as any forward movement of the hand prior to the actual start of the test could be detected by the Kinect as the start time. Another factor which could possibly have affected the Kinect times was changes in the level of lighting in the room, since the RGB values of the color images were dependent on this factor. This issue may be solved by relying solely on the depth output of the Kinect sensor, as this output is not affected by the level of lighting in the room (except from direct sunlight). The results from the analysis of the agreement between the therapist times and the Kinect times described in the present study were comparable to the results presented in [10, 11] and [13], all generally showing that the automated systems estimated task durations to be shorter compared to the estimates made by therapists. The statistically significant biases of the differences between the ground truth and therapist times (negative biases for end times and positive biases for total times) ranged from −0.18 to 0.70 s, whereas the only significant bias of the ground truth and Kinect times was a positive bias of 0.20 s (end time bias). From these results, it is clear that the accuracy of the Kinect times is better than the accuracy of the therapist times. Considering the lower accuracy of the therapist times (compared to the accuracy of the Kinect times) and the sizes of the biases of the end times, human reaction time seems to be an evident explanation. The bias of the therapist and Kinect times can easily be handled by adding an offset to future test results, whereas the variability of the two methods, indicated by their agreement with ground truth times, cannot be handled by adding a time offset. Therefore, the lower variability demonstrated by the Kinect based system is a promising result, showing that this type of technology might be useful for assessment of motor function in future tele-rehabilitation settings. Although, the variability of the Kinect based system is lower than the variability of the therapist, the magnitude of the variability of both methods is not critical, when considering average changes in test scores from other studies on stroke patients [4, 19]. However, the main focus of the paper was to show that the Modified Jebsen Test of Hand Function can be automatized and that the precision of this automated version of the test is comparable to the precision of a therapist operating a stop-watch. Thus, automatized motor function tests could be used as a tool by different therapists, to minimize variability when doing repeated tests on the same patient. In agreement with a number of other studies [10, 11, 13], the results from the present study shows that kinematic sensors can be used to automate timed motor function tests, producing timing estimates that are close to ground truth results and/or manually recorded results (<1 s).

### Assessment of upper extremity motor function in stroke patients

Changes in motor ability following stroke can occur as a result of either motor recovery (restoration of movements that were in place before the stroke) or compensation (movements are performed in an alternative manner) [20]. Patients who have suffered from a stroke often develop altered movement strategies in an attempt to perform activities of daily living [21]. Although compensatory movement strategies result in functional gains, these new movement strategies might be inappropriate on the long term, leading to pain and inhibition of motor recovery [22]. To ensure an optimal rehabilitation strategy, the assessment of stroke patients should include some form of qualitative assessment of the movement patterns to capture inappropriate movement patterns, e.g. a therapist providing verbal and physical feedback to the patient during training. Motor function tests as e.g. the MJT, Nine Hole Peg Test [9], and Box and Blocks Test [8] are functional scales that do not assess the motor patterns of the subject during completion of the test. The underlying physiological nature of outcome improvement in these tests can therefore not be identified from the test results only, but would require a complementary qualitative assessment. Alternatively, the Microsoft Kinect could possibly be used to estimate the quality of movements or detect compensatory movements [23].

In the present study, the MJT was administered twice to each subject within the same session. The ground truth MJT times measured in the present study were in a range between times measured in healthy subjects [4] and other stroke subjects, e.g. [24]. The results also revealed significant improvements in performance in the second assessment. In a previous study the MJT was administered twice (average time between tests was ~10 days) to 23 individuals with acquired neurological disorders and found no changes in performance [14]. The practice effect found in the present study is therefore most likely related to functional changes that are specifically related to the MJT tasks and thus the functional changes are not a sign of general functional improvement. Motor function tests should therefore not be administered too close in time in order to avoid inducing test specific practice effects.

### Perspectives on automated assessment of upper extremity motor Function in Stroke Patients

In the present study we showed that it is possible to automate the MJT by use of a Kinect sensor. A number of recent studies have also demonstrated solutions for automating existing validated motor function tests [10, 11, 13]. Automation of motor function tests holds great potential as it not only increases the objectivity of the tests but also possibly makes the tests applicable at remote sites without need for direct supervision, thereby decreasing pressure on clinicians. This could potentially help solving one of the challenges associated with provision of tele-rehabilitation services, which is to conduct remote assessment of patients [25]. However, an important challenge remains for tele-rehabilitation systems that are to be used without direct supervision. This challenge relates to the validation of the quality of the data collected by the system, e.g. poor performance in a test might not necessarily be related to the patient’s training compliance, but could as well be related to lack of motivation [26], pain due to inappropriate movement strategies [22], fatigue or erroneous recordings. Combining automated assessment of motor function with techniques monitoring the patient’s facial expressions and/or body language, e.g. by use of social signal processing [27], could possibly enhance the validity of the recorded data, as this type of information could help determine whether a patient was motivated during a training or test session. Besides the use of automated testing for remote testing of stroke patients, the concept may also be used to test more patients simultaneously (using multiple test systems), which would also decrease pressure on clinicians.

## Conclusion

The aim of the current study was to design and test a Kinect based system for automatic evaluation of the Modified Jebsen Test of Hand Function (MJT). The MJT was administered twice to 11 stroke patients with hand function deficits. The test was performed manually by a therapist using a stopwatch and automatically by use of a Kinect.

The results from the test showed that it is possible to use a Kinect based system to automate the MJT. Overall, the agreement between the ground truth results and the Kinect based evaluations was better than the agreement between the ground truth results and the manual assessment performed by the therapist. Results from the test-retest of the MJT demonstrated significant improvements in the performance time, indicating a practice effect.

## Abbreviations

BBS: Berg Balance Scale
D: Dominant hand
F: Female
Kinect: Microsoft Kinect sensor
LoA: Limits of Agreement
M: Male
MGT: Median ground truth
MJT: Modified Jebsen Test of Hand Function
N: Non-dominant hand
RGB: Red, green, blue
SMWT: Six Minutes Walk Test
WMFT: Wolf Motor Function Test
